# Neutrophil heterogeneity under IL-17A deficiency in experimental periodontitis

**DOI:** 10.3389/fimmu.2026.1963694

**Published:** 2026-10-13

**Authors:** Zhongyuan Tang, Renjie Hu, Luying Zhu, Jing Hu, Fan Xiao, Lijian Jin, Liwei Lu, Yanqi Yang

**Affiliations:** 1 Division of Paediatric Dentistry and Orthodontics, Faculty of Dentistry, The University of Hong Kong, Hong Kong, Hong Kong SAR, China; 2 Department of Pathology and Shenzhen Institute of Research and Innovation, The University of Hong Kong, Hong Kong, Hong Kong SAR, China; 3 Division of Periodontology and Implant Dentistry, Faculty of Dentistry, The University of Hong Kong, Hong Kong, Hong Kong SAR, China

**Keywords:** cell–cell communication, interleukin−17A, neutrophil heterogeneity, periodontitis, single-cell RNA sequencing

## Abstract

Periodontitis is a dysbiotic inflammatory disease characterized by progressive periodontal destruction, in which neutrophils play a central but functionally diverse role. IL-17A is closely linked to neutrophil-mediated host responses; however, how IL-17A deficiency reshapes neutrophil heterogeneity during periodontitis remains unclear. Here, we established a *Porphyromonas gingivalis*–induced experimental periodontitis model in wild-type and IL-17A knockout mice and integrated micro-CT, histology, 16S rRNA sequencing, and single-cell RNA sequencing to define the consequences of IL-17A loss in the periodontal microenvironment. IL-17A deficiency exacerbated alveolar bone loss and osteoclast accumulation and was associated with altered oral microbial composition consistent with dysbiosis. Single-cell profiling revealed marked remodeling of the immune landscape, with impaired neutrophil accumulation and altered neutrophil subset composition in IL-17A-deficient periodontal tissues. Neutrophils from IL-17A knockout mice showed reduced inflammatory chemokine/cytokine programs and a shift toward antimicrobial granule-associated, extracellular matrix-responsive, and TGF-β/THBS-linked transcriptional states. Trajectory, transcriptional regulatory, and cell–cell communication analyses further demonstrated that IL-17A deficiency disrupted neutrophil state transitions and attenuated neutrophil-centered CCL, CXCL, TNF, SELPG, and ICAM signaling while enhancing matrix-associated communication pathways. Together, these findings identify IL-17A deficiency as a driver of neutrophil reprogramming and aggravated periodontal destruction and indicate an association with microbial dysbiosis, providing single-cell-level insight into the IL-17A–neutrophil axis in dysbiotic periodontitis.

## 1 Introduction

Periodontal disease is a common chronic inflammatory condition and represents a major burden to global health. Periodontitis is initiated by dysbiotic dental biofilms and is characterized by progressive destruction of the gingiva, periodontal ligament, and alveolar bone, ultimately leading to tooth loss (1, 2). Beyond local tissue destruction, advanced periodontitis is closely associated with multiple systemic diseases, including cardiovascular diseases and diabetes, underscoring its broader clinical significance (3).

Among periodontal microorganisms, the anaerobic Gram-negative *Porphyromonas gingivalis* (*P. gingivalis*) has been extensively implicated in the pathogenesis of chronic periodontitis (4–8). As a key pathogen, *P. gingivalis* can reshape the oral microbial community and disrupt host–microbiota interactions, thereby promoting microbial dysbiosis, amplifying inflammation, and accelerating alveolar bone resorption (6–10). Under physiological conditions, host immune signals contribute to periodontal homeostasis by coordinating antimicrobial defense, inflammatory resolution, and bone remodeling. In susceptible hosts, however, disruption of immune regulation facilitates the transition from reversible gingival inflammation to irreversible periodontitis, which is characterized by persistent inflammation and progressive bone destruction (11). Thus, periodontitis progression reflects not only the presence of pathogenic bacteria but also dysregulated immune–microbial interactions within the periodontal microenvironment.

Interleukin-17A (IL-17A), a signature cytokine of the IL-17 family, is widely recognized for its pleiotropic roles in mucosal immunity, inflammation, and bone-related immune regulation (12, 13). In periodontitis and other inflammatory bone diseases, IL-17A has been implicated in host defense and inflammatory regulation, particularly through its effects on neutrophil recruitment and activation (14–17). However, the role of IL-17A in periodontal inflammation appears to be context dependent. While excessive IL-17A-driven inflammation may contribute to tissue damage under certain conditions, impaired IL-17A signaling may compromise antimicrobial defense and disturb immune homeostasis in infection-associated settings. Therefore, defining the cellular and molecular consequences of IL-17A deficiency is essential for understanding how IL-17A shapes periodontal immune responses during bacterial challenge.

Neutrophils are continuously generated from myeloid precursors in the bone marrow and enter the circulation as short-lived effector cells before migrating into peripheral tissues, where they participate in microbial clearance, inflammatory amplification, tissue remodeling, and subsequent resolution (18, 19). Traditionally considered a relatively homogeneous innate immune population, neutrophils are now increasingly recognized as highly heterogeneous and plastic cells. Their heterogeneity is shaped by developmental maturation along the bone marrow-derived neutrotime continuum, transcriptional diversification in circulation, and microenvironmental cues within inflamed tissues, collectively supporting context-dependent functions in homeostasis and disease (20). In periodontal tissues, neutrophils represent a dominant immune cell population and are essential for maintaining the balance between microbial containment and inflammatory tissue injury. Dysregulated neutrophil recruitment or function may therefore contribute to both microbial dysbiosis and periodontal destruction.

Accumulating evidence suggests that neutrophil heterogeneity and microenvironment-specific signaling critically influence the progression and outcome of periodontitis (21). Nevertheless, whether IL-17A deficiency alters neutrophil recruitment, functional state transitions, and neutrophil-centered intercellular communication during periodontitis remains incompletely understood. In particular, it is unclear how loss of IL-17A reshapes neutrophil transcriptional programs under sustained *P. gingivalis* challenge and how these changes are linked to microbial dysbiosis and periodontal bone destruction. To address this gap, we established a *P. gingivalis*–induced experimental periodontitis model in IL-17A knockout mice and performed single-cell RNA sequencing of periodontal tissues. By integrating neutrophil subclustering, functional enrichment, trajectory inference, transcriptional regulatory analysis, and cell–cell communication analysis, we aimed to define IL-17A deficiency-associated neutrophil heterogeneity and elucidate the immune regulatory alterations underlying aggravated periodontal inflammation.

## 2 Materials and methods

### 2.1 Animal models

Eight-week male C57BL/6N WT and IL-17A KO mice (n = 6/subgroup) underwent live *P. gingivalis*–induced experimental periodontitis (PD) for imaging, histology, and microbiome analyses (WT control, IL-17A KO control, WT periodontitis, IL-17A KO periodontitis). *P. gingivalis* strain W83 was cultured anaerobically on horse blood agar plates in trypticase soy broth supplemented with hemin and vitamin K. For ligature placement, 5-0 silk sutures were tied around the cervical region of bilateral maxillary molars for 14 days. Ligatures were soaked in *P. gingivalis* (10^9^ CFU/mL) and re-soaked twice weekly to maintain stimulation. Prior to the procedure, mice were anesthetized with ketamine (80 mg/kg, i.p.) and xylazine (10 mg/kg, i.p.) to minimize stress and movement. On Day 14, the mice were euthanized with sodium pentobarbitone (150 mg/kg, i.p.), and the maxillae were subsequently dissected for further analysis.

In a separate cohort for single-cell RNA-seq, periodontal tissues were harvested from C57BL/6N WT and IL-17A KO mice subjected to either live P. gingivalis infection or sham treatment (healthy control) (n = 8 per subgroup). Tissues from 8 mice were pooled per subgroup to generate one single-cell suspension and one scRNA-seq library per condition (four libraries total: WT control, IL-17A KO control, WT periodontitis, IL-17A KO periodontitis), using standardized dissections around the maxillary second molar to ensure anatomical consistency (22). Sample sizes were determined with reference to prior studies employing pooled periodontal single-cell preparations (23). Between-group inferences from scRNA-seq are presented descriptively and interpreted with caution given the library-level replication structure.

All animal experiments were conducted under specific pathogen-free conditions and approved by the Committee on the Use of Live Animals in Teaching and Research (CULATR No. 4639-18). IL-17A KO mice were kindly provided by Dr Yoichiro Iwakura at The Institute of Medical Science, The University of Tokyo, Japan.

### 2.2 Micro-CT

Micro-CT analysis was performed using a SkyScan 1276 system (Bruker) with a voxel size of 20 μm. Mice were anesthetized before scanning, and scans were acquired on Day 14 of disease progression. Alveolar bone loss was quantified as the linear distance from the cementoenamel junction (CEJ) to the alveolar bone crest (ABC) measured at the mesiobuccal aspect of the maxillary second molar, using the same anatomical location for all animals.

### 2.3 Histological and immunostaining analyses

Maxillae were fixed in 10% formalin, decalcified in EDTA, embedded in paraffin, and sectioned at 4 μm. Sections were stained with hematoxylin and eosin for histological evaluation. TRAP staining was performed to identify multinucleated osteoclasts (≥3 nuclei). For immunofluorescence, deparaffinized sections underwent heat-induced epitope retrieval, were blocked, and incubated with primary antibodies against CCL3 and LY6G (Servicebio), followed by fluorescent secondary antibodies. Nuclei were counterstained with DAPI. Representative images were acquired at 20× and 60×; quantification was performed at 60× using standardized fields around the mesial side of the maxillary second molar, with blinded assessment (n = 6 per group).

### 2.4 Oral microbiota analysis

Oral microbiota was profiled by 16S rRNA V3–V4 amplicon sequencing on a DNBSEQ-G400 (MGI/BGI; paired-end 2 × 300 bp). Reads were quality-filtered (cutadapt v2.6; Phred <20 over 25 bp trimmed; short/N/low-complexity reads removed), merged (FLASH v1.2.11), and de-chimerized (UCHIME v4.2.40). OTUs were constructed with UPARSE/USEARCH v7 at 97% identity; taxonomy was assigned using the RDP Classifier (confidence 0.6) against Greengenes v201305. Per-sample depth was 39, 341–74, 937 raw pairs; beta-diversity analyses used rarefied OTU tables (QIIME v1.80). Alpha diversity (sobs, Chao1, ACE, Shannon, Simpson, Good’s coverage; mothur v1.31.2) was compared by Wilcoxon or Kruskal–Wallis tests (P < 0.05). Beta diversity (Bray–Curtis; unweighted/weighted UniFrac) was evaluated with ANOSIM/MRPP and, where applicable, PERMANOVA (vegan). Differential taxa were identified by LEfSe (Kruskal–Wallis α = 0.05; pairwise Wilcoxon α = 0.05; LDA ≥ 2.0).

### 2.5 Single-cell suspension preparation

Periodontal tissues including the gingiva, alveolar bone and periodontal ligament, from eight mice per sample were enzymatically dissociated into single-cell suspensions using collagenase I and dispase II. After filtration, red blood cells were lysed. Samples with viability >90% and concentrations of 800–1, 500 cells/μL were processed for scRNA-seq.

### 2.6 ScRNA-seq analysis

Single-cell libraries were prepared on the 10× Genomics Chromium platform and sequenced on an Illumina NovaSeq. Reads were aligned to the mm10 reference with Cell Ranger. Data were processed in Seurat with standard QC (cells with fewer than 300 detected genes or more than 20% mitochondrial reads removed). Batch effects were corrected using Harmony, followed by PCA and UMAP for dimensionality reduction and clustering. Differential expression was assessed with the Wilcoxon rank-sum test using Seurat::FindMarkers, and adjusted P values were calculated with Benjamini–Hochberg false discovery rate (FDR). Gene co-expression modules were identified using WGCNA. Pathway activity was evaluated with GOBP, GSVA, and AUCell, and differential pathway enrichment was analyzed using generalized linear models. RNA velocity was inferred with scVelo, pseudotime trajectories were reconstructed with Monocle2, and GO enrichment was performed with clusterProfiler. Cell–cell communication was analyzed with CellChat to infer ligand–receptor interactions, and NicheNet was used to prioritize ligands regulating neutrophil gene expression in WT and IL-17A KO mice.

### 2.7 Statistical analysis

Group differences in bone loss were assessed by two-way ANOVA with factors Genotype (WT, IL-17A KO), Condition (control, periodontitis), and their interaction. Where noted, simple-effects comparisons within a condition or genotype were evaluated using Welch’s t-tests. A two-tailed *p* < 0.05 was considered statistically significant. For single-cell RNA-seq, differentially expressed genes were identified with Seurat::FindMarkers (Wilcoxon rank-sum), and adjusted P values were calculated using Benjamini–Hochberg FDR; adjusted P (q) values are reported.

## 3 Results

### 3.1 IL-17A deficiency aggravates *P. gingivalis*-induced periodontitis and regulates oral microbiota composition

WT and IL-17A KO mice were subjected to the *P. gingivalis*-induced periodontitis and CEJ–ABC distance assessed by micro-CT. A significant inter-genotype difference was observed during progression of periodontitis (**Figure 1A**). H&E staining confirmed a greater abundance of resorption lacunae and more disorganized trabecular architecture in infected IL-17A KO mice (**Figure 1C**). TRAP staining demonstrated significantly increased multinucleated osteoclast-like cells on alveolar bone surfaces in IL-17A KO mice (**Figure 1D**).We next evaluated the impact of IL-17A on the composition of the oral microbiota. 16S rRNA profiling identified 330 OTUs, of which 89.4% were shared between groups (**Supplementary Figure 1A**). PCoA and α-diversity indices revealed a shift in community structure with IL-17A deficiency (**Supplementary Figures 1B, C**). At the phylum level, IL-17A KO mice exhibited increased *Proteobacteria* and *Bacteroidetes*, and decreased *Firmicutes* (**Figure 1B**). At the genus level, *Porphyromonas* and *Bacteroides* were enriched while *Staphylococcus*, *Lactobacillus* and *Olsenella* were reduced (**Supplementary Figure 1D**; **Supplementary Table 6**). LEfSe identified *Escherichia* as significantly enriched in IL-17A KO mice (LDA>4.8; **Supplementary Figure 1E**).

**Figure 1:**
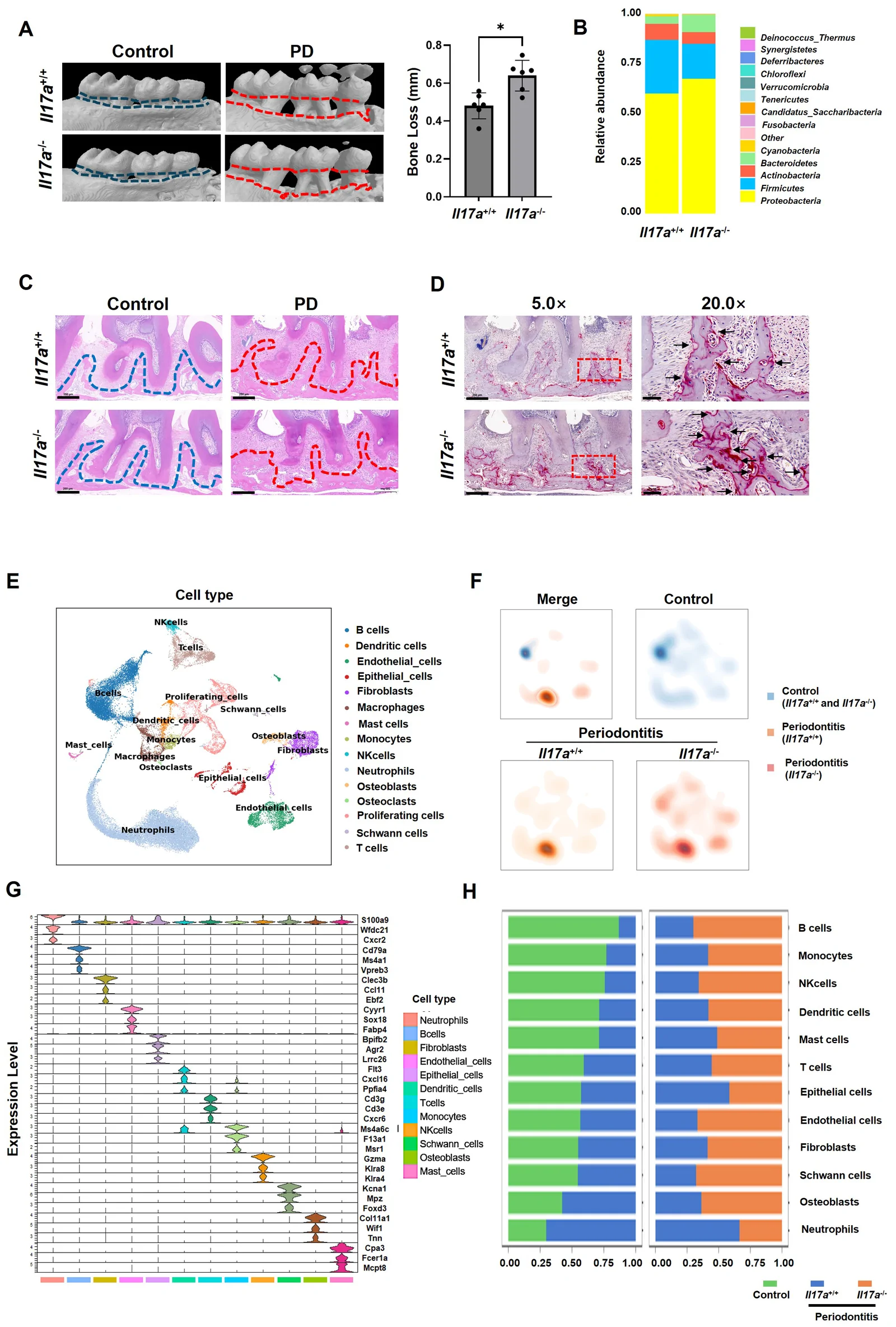
IL-17A deficiency aggravates alveolar bone loss in experimental periodontitis and regulates oral microbiota composition and single-cell transcriptomic landscape of periodontal tissues. **(A)** Representative micro-computed tomography (micro-CT) images of maxillary alveolar bone in WT and IL-17A KO mice under control and periodontitis conditions. Quantification of alveolar bone loss, measured as the distance from the cementoenamel junction (CEJ) to the alveolar bone crest (ABC). **(B)** Relative abundance of major oral bacterial phyla in WT and IL-17A KO mice under periodontitis conditions. **(C)** Representative hematoxylin and eosin (H&E) staining of maxillary periodontal tissues from WT and IL-17A KO mice; scale bars, 200 μm (5×). **(D)** Representative TRAP staining of maxillary periodontal tissues from WT and IL-17A KO mice; scale bars, 200 μm (5×) and 50 μm (20×). **(E)** Uniform Manifold Approximation and Projection (UMAP) showing major cell populations identified in periodontal tissues; UMAP colored by experimental groups (WT healthy controls; periodontitis: WT and IL-17A KO). **(F)** Cell density distribution on the UMAP colored by experimental groups. **(G)** Violin plots showing representative marker gene expression across identified cell types. **(H)** Relative proportions of cell populations in control versus WT periodontitis, and in WT versus IL-17A KO under periodontitis.

### 3.2 IL-17A regulates immune cell composition in periodontium

Histological assessment also showed that inflammatory cell infiltration in the gingiva and periodontal ligament was substantially lower in IL-17A KO mice (**Figure 1C**). To obtain a preliminary profile of the cellular landscape in *P. gingivalis*-infected periodontal tissue and examine its relationship with IL-17A, we performed scRNA-seq on samples from WT and IL-17A KO mice after infection. Periodontal tissues were dissected, enzymatically digested, and processed using droplet-based scRNA-seq.

After sequencing, 44, 228 cells were captured, with 22, 485 genes detected overall. Data preprocessing was carried out with the Seurat pipeline. The median number of genes detected per cell (nFeature_RNA) was 1, 339, and cells with mitochondrial gene expression exceeding 20% were filtered out. Following quality control, the 3, 000 most variable genes were selected for downstream analysis. Dimensionality reduction via uniform manifold approximation and projection (UMAP) was used to visualize the cellular composition (**Figure 1E**).

Based on the expression of canonical cell-type marker genes, cells were classified into thirteen distinct populations, including neutrophils (*S100a9*), B cells (*Cd79a*), fibroblasts (*Ebf2*), endothelial cells (*Sox18*), epithelial cells (*Bpifb2*), dendritic cells (*Flt3*), T cells (*Cd3e*), monocytes and macrophages (*Msr1*), NK cells (*Klra4*), Schwann cells (*Mpz*), osteoblasts (*Col11a1*), mast cells (*Fcer1a*), and proliferating cells. The top three highly expressed genes for each cell type were identified and compared, and the expression patterns of representative signature genes for each defined population are shown in **Figure 1G**.

We next compared the density distribution maps and relative proportions of each cell cluster across different experimental groups (**Figure 1H**). In both periodontitis groups, the most dominant immune cell population was neutrophils (**Figure 1F**). The absence of IL-17A substantially alters the immune cell composition within the inflamed periodontium. Compared with the IL-17A KO group, the WT group was characterized by a higher proportion of neutrophils, whereas the proportions of other immune cells were lower (**Figure 1F**).

### 3.3 IL-17A influences neutrophil heterogeneity and functional adaptations in periodontitis

Neutrophils are known to play key roles in tissue homeostasis, inflammatory regulation, and tissue injury (24, 25). In this dataset, neutrophils represented the most abundant immune cell type overall and also exhibited the most prominent changes following IL-17A knockout (**Figure 1F**). Given this, we performed further UMAP-based subclustering of the neutrophil compartment to characterize distinct functional subsets in the periodontal tissue after *P. gingivalis* challenge.

After removing doublets (cluster 5), neutrophils originally identified in **Figure 2A** separated into five transcriptionally distinct subclusters: Ccl3^+^ neutrophils (cluster 0), Fosb^+^ neutrophils (cluster 1), Tgfbi^+^ neutrophils (cluster 2), Ltf^+^ neutrophils (cluster 3), and Retnlg^+^ neutrophils (cluster 4) (**Figure 2B**). Total neutrophil numbers, as well as counts within each subcluster, were higher in WT mice than in IL-17A KO mice. Relative to WT mice, IL-17 KO mice exhibited reductions in both overall cellular abundance and subpopulation-specific cell counts within clusters 0, 1, 2, and 3 (**Figure 2C**). Marker genes defining these ten neutrophil subpopulations are listed in **Supplementary Table S1**. Of note, clusters 1 and 2 were more abundant in periodontal tissues from WT mice, whereas clusters 3, and 4 were markedly enriched in IL-17A KO mice (**Figure 2D**). To further validate these findings, immunofluorescence staining was performed on periodontal tissues from WT and IL-17A KO mice. Periodontitis induction led to a pronounced accumulation of LY6G^+^ neutrophils in WT mice, accompanied by a significant increase in the proportion of CCL3^+^LY6G^+^ neutrophils. In contrast, this increase was markedly attenuated in IL-17A KO mice, suggesting that IL-17A contributes to neutrophil-associated CCL3 expression in periodontal inflammation. (**Figure 2E**).

**Figure 2:**
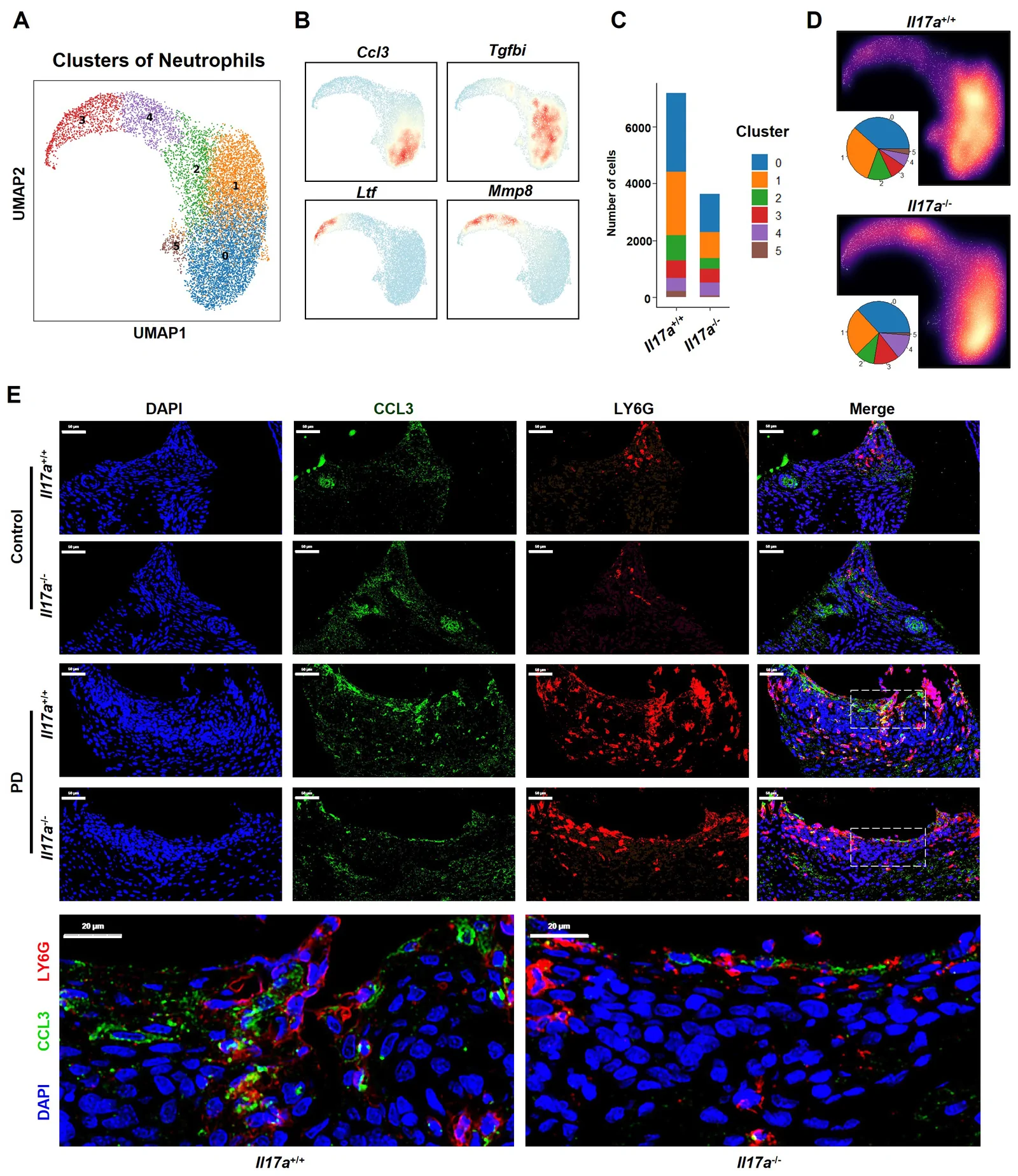
IL-17A shapes neutrophil heterogeneity in experimental periodontitis. **(A)** UMAP embedding of neutrophils showing distinct subclusters. **(B)** Marker gene expression overlaid on the UMAP of neutrophil subclusters. **(C)** Relative abundance of neutrophil subclusters in WT and IL-17A KO mice with periodontitis. **(D)** Spatial density and proportional composition of neutrophil subclusters; color intensity denotes local cell density (red = higher). **(E)** Representative immunofluorescence images of periodontium with double labeling for DAPI (blue), CCL3 (green), and LY6G (red); scale bars, 50 μm (20×) and 20 μm (60×).

Functional annotation based on gene programs indicated that cluster 0 corresponds to Ccl3+ inflammatory neutrophils with high expression of pro-inflammatory cytokines/chemokines (*Ccl3*, *Tnf*, *Il1a*, *Ccl4*, and *Cxcl3*). Cluster 2 corresponds to Tgfbi+ repair-associated neutrophils enriched for tissue-remodeling and matrix-interaction genes (*Tgfbi*, *Timp2*, *Jaml*, and *Wfdc17*). Cluster 3 represents granule-rich antimicrobial neutrophils (*Chil3*, *Camp*, *Ltf*, and *Ngp*). Cluster 4 represents infiltrating/ECM-responsive neutrophils with elevated *Retnlg* and *Mmp8*. Cluster 1 is characterized as apoptotic-like neutrophils based on increased mitochondrial transcripts (*mt-Nd1/2/3/4l/5* and *mt-Cytb*) (26–28).

The weighted gene co-expression network analysis (WGCNA) was performed to further characterize the functional profiles of individual neutrophil subclusters. Four unique gene co-expression modules were delineated and designated as NEUT M1 (turquoise), NEUT M2 (blue), NEUT M3 (yellow), and NEUT M4 (brown) (**Figure 3A**). The NEUT M1 module included genes such as Ltf, Camp, Ngp, Lyz2, and Cyba. NEUT M2 contained Cxcl2, Nlrp3, Clec4d, Srg1, and Plek. NEUT M3 comprised Thbs1, Homer1, Ankrd2, Selenon, and Coq10b. NEUT M4 was enriched for Zyx as well as members of the Rpl/Rps gene families (**Figure 3B**). Module expression analysis across neutrophil subclusters revealed that NEUT-M2 genes were predominantly expressed in Ccl3^+^ neutrophils (cluster 0); NEUT-M1 genes were mainly enriched in Ltf^+^ neutrophils (cluster 3) and Retnlg^+^ neutrophils (cluster 4); NEUT-M3 genes were primarily expressed in Retnlg^+^ neutrophils (cluster 4); and NEUT-M4 genes were mainly expressed in Retnlg^+^ neutrophils (cluster 4) and Tgfbi^+^ neutrophils (cluster 2) (**Figure 3C**). Comparison of module expression between experimental groups demonstrated that NEUT-M2 and NEUT-M4 were preferentially upregulated in WT mice, whereas NEUT-M3 and NEUT-M1 were predominantly upregulated in the IL-17A KO group (**Figure 3D**).

**Figure 3:**
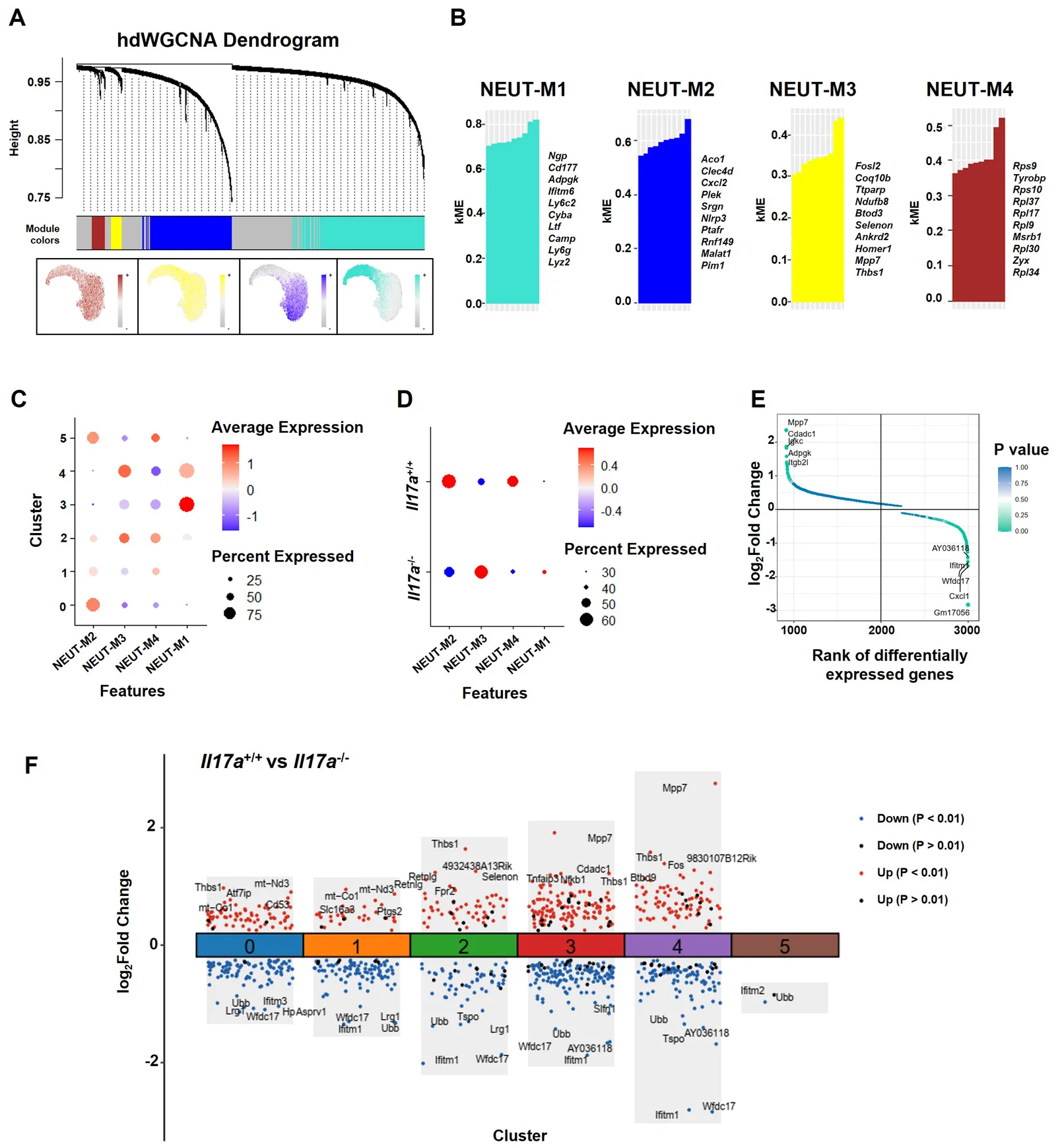
IL-17A alters neutrophil functional states in experimental periodontitis. **(A)** Weighted gene co-expression network analysis (WGCNA) identifying four neutrophil co-expression modules. **(B)** Top 10 hub/co-expressed genes within each WGCNA module. **(C, D)** Bubble plots showing module expression across neutrophil subpopulations and in WT versus IL-17A KO. **(E)** Waterfall plot of average log2 fold-changes for differentially expressed genes (DEGs) in neutrophils (WT vs IL-17A KO). **(F)** Scatter plot of average log2 fold-changes showing up- and down-regulated DEGs across neutrophil subclusters in IL-17A KO relative to WT during periodontitis. Test: Wilcoxon rank-sum, BH-FDR (q). Significance: adjusted P < 0.01 in red or blue; adjusted P ≥ 0.01 in black.

We next analyzed global differentially expressed genes (DEGs) in neutrophils between healthy WT and healthy IL-17A KO mice, as well as between WT and IL-17A KO mice under periodontitis conditions (**Figure 3E**). Compared with healthy controls, neutrophils from WT mice with periodontitis exhibited characteristic upregulation of *Cxcl1*, *Wfdc17*, *Ifitm1*, *Ubb*, and *Igfbp6*, whereas neutrophils from IL-17A KO mice showed increased expression of *Cdadc1*, *Adpgk*, *Itgb2l*, *Nt5e*, and *Thbs1* (**Supplementary Tables S2–S5**). DEGs between healthy WT and healthy IL-17A KO mice were also identified (**Supplementary Figure 2A**). Mapping these DEGs onto individual neutrophil subclusters revealed broadly consistent expression patterns across clusters, in agreement with the global trends—most notably, increased expression of *Ifitm1* and *Wfdc17* in WT mice and elevated expression of *Thbs1* in the IL-17A KO group. Notably, the upregulation of Thbs1 in the IL-17A KO group was consistent with the overall expression trend of genes within the NEUT-M3 module identified by WGCNA. These differences were most evident in cluster 4, followed by clusters 3 and 2, and were relatively less pronounced in clusters 1 and 0 (**Figure 3F**).

### 3.4 IL-17A modulates neutrophil state transitions and trajectory dynamics in periodontitis

Neutrophils display substantial functional plasticity in response to inflammatory cues within the local tissue microenvironment, giving rise to heterogeneous subpopulations with distinct transcriptional programs (29). In the context of *P. gingivalis*–driven periodontitis, we sought to determine whether IL-17A influences the progression of these neutrophil states over time. To this end, RNA velocity analysis was performed using scVelo to infer directional state transitions and reconstruct inflammation-associated fate trajectories (**Figure 4A**). Monocle2 pseudotime analysis was employed to delineate three distinct neutrophil states (**Figure 4B**).

**Figure 4:**
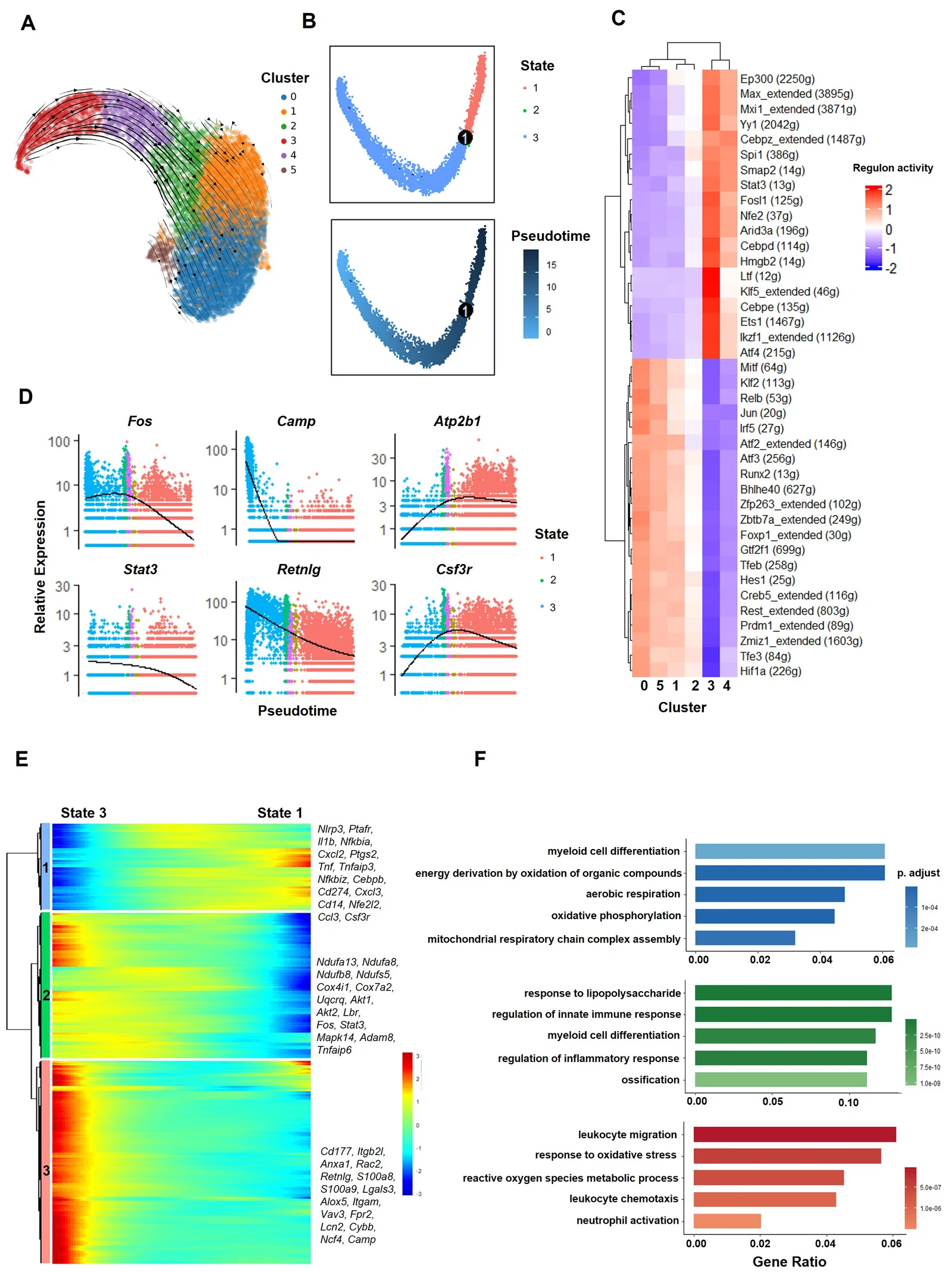
Neutrophil state transitions and trajectory dynamics in periodontitis are modulated by IL-17A. **(A)** UMAP embedding of neutrophils with the same color annotations, overlaid with streamlines denoting RNA velocity. **(B)** Upper panel, neutrophil trajectory states during inflammation, showing three discrete stages; lower panel, neutrophil pseudotime along the inflammatory trajectory. **(C)** Heatmap showing SCENIC regulon activity, identifying highly active transcription factors across neutrophil subclusters. **(D)** Expression dynamics of selected marker genes along pseudotime, progressing from State 3 to State 1. **(E)** Heatmap of scaled expression for differentially expressed genes between two states, grouped into three major gene clusters (labels at left) that vary as a function of pseudotime; representative genes for each cluster are highlighted along the right margin; from left to right, the heatmap follows a continuous lineage path from State 3 to State 1. **(F)** Pathway enrichment analysis associated with three gene clusters.

State 1 primarily aligned with the Ccl3^+^ neutrophil subcluster (cluster 0), whereas State 3 consisted mainly of Ltf^+^ (cluster 3) and Retnlg^+^ neutrophils (cluster 4) (**Figure 4b**). These results were consistent with SCENIC analysis of subcluster-specific transcription factor activity, which showed stronger STAT3 regulon activity in clusters 3 and 4 (**Figure 4C**), as well as increased *Stat3* expression along pseudotime in State 3 (**Figure 4D**). Because most neutrophils were distributed in States 1 and 3, we next compared the gene expression profiles distinguishing these two states and performed Gene Ontology biological process (GOBP) enrichment analysis (**Figure 4E**).

Genes in the green module, including *Fos*, *Stat3*, *Retnlg*, and *Camp*, whose expression rose progressively toward State 3, were significantly enriched for GOBP terms such as Response to Lipopolysaccharide and Regulation of Innate Immune Response. In the state 1, genes in the red module, such as *Csf3r* and *Atp2b1*, were enriched for processes related to Leukocyte Migration, Leukocyte Chemotaxis, and Neutrophil Activation, as well as for Response to Oxidative Stress and Reactive Oxygen Species Metabolic Process. The functional patterns are consistent with a pro-inflammatory phenotype characteristic of Ccl3^+^ neutrophil subcluster (**Figure 4F**).

### 3.5 IL-17A remodels neutrophil-centered cell–cell communication networks in periodontitis

Next, we performed CellChat analysis on all identified cell types from WT and IL-17A KO mice by quantifying ligand–receptor interaction strengths to investigate intercellular communication under the influence of IL-17A in the inflamed periodontium. We found that neutrophil is the most active communicating cell type among all identified cell types in both incoming and outgoing signals. WT mice showed weaker outgoing interaction strengths in fibroblasts, endothelial cells, and osteoblasts, but stronger overall signals in neutrophils compared with IL-17A KO mice (**Supplementary Figure 2B**).

The heatmap in **Figure 5B** showed clear differential interaction-strength between genotypes. The ligands from fibroblasts, endothelial cells, osteoblasts, and neutrophils themselves substantially regulate neutrophil activity. At the neutrophil pathway level, WT mice showed stronger interaction strength of CCL, CXCL, TNF, SELPG, and ICAM signaling, but weaker interaction strength of COLLAGEN and THBS signaling, compared to IL-17A KO mice. COLLAGEN signaling was also consistently upregulated in endothelial cells, fibroblasts, and osteoblasts of IL-17A KO mice, followed by increased THBS, LAMININ, and FN1 pathway activity. Besides, we found that neutrophils functioned primarily as senders of THBS signaling, while endothelial cells and fibroblasts acted as the main recipients of these signalings (**Figure 5C**; **Supplementary Figure 2B**).

**Figure 5:**
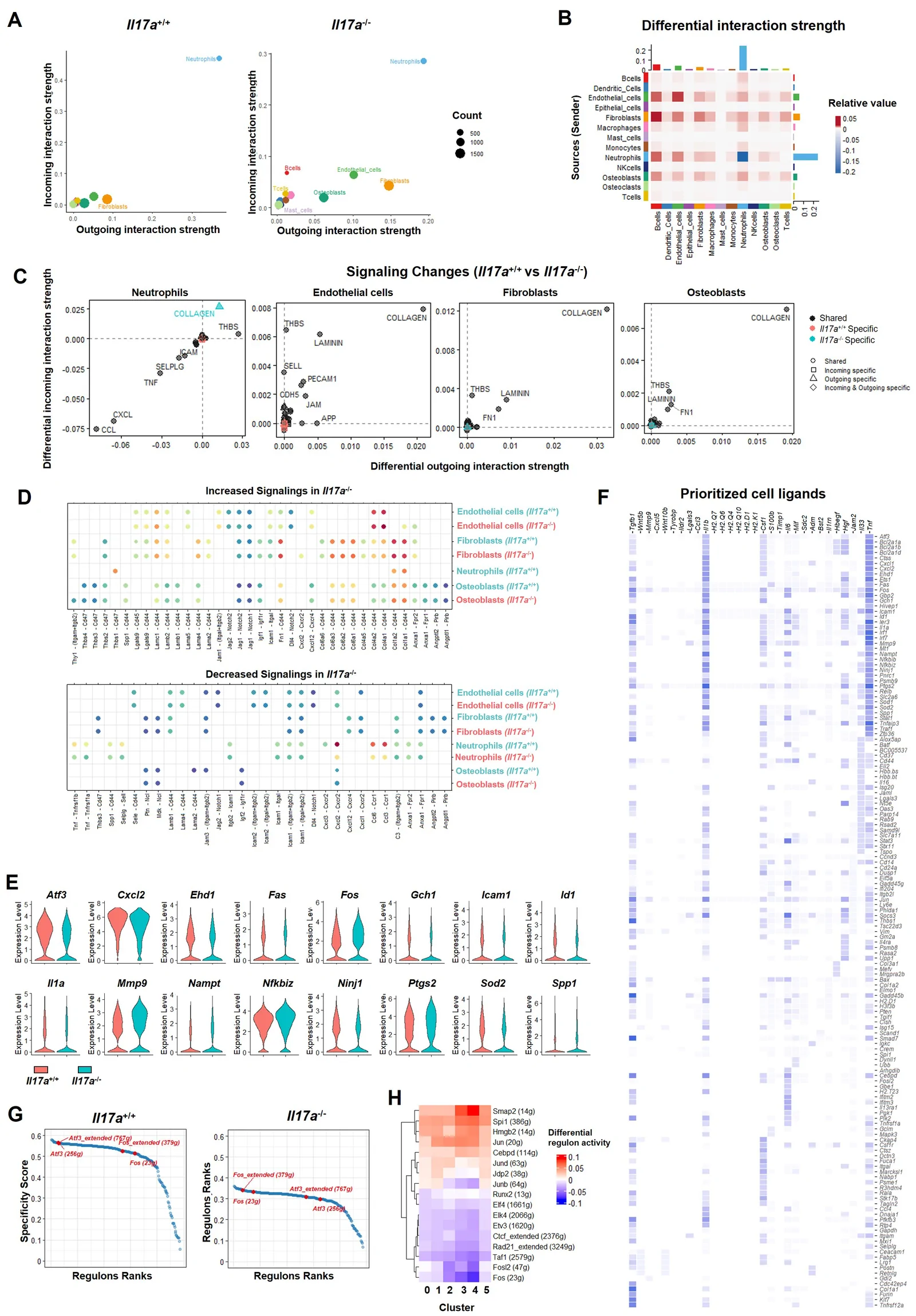
Neutrophil-centered intercellular communication in periodontitis is remodeled by IL-17A. **(A)** Scatter plots showing significant changes in outgoing and incoming interaction strength across cell populations in WT and IL-17A KO mice with periodontitis, analyzed by CellChat. **(B)** Heatmap of differential interaction strength among cell populations between WT and IL-17A KO. **(C)** Specific signaling changes in the top four cell populations ranked by interaction strength between WT and IL-17A KO. **(D)** Dysfunctional ligand–receptor pairs targeting neutrophils (receiving cells), originating from endothelial cells, fibroblasts, osteoblasts, and neutrophils (sending cells). **(E)** Violin plots showing representative genes involved in intracellular signaling and transcriptional regulation with differential expression between WT and IL-17A KO mice. **(F)** Heatmap showing NicheNet-inferred effects of ligands from endothelial cells, fibroblasts, osteoblasts, and neutrophils on neutrophil intracellular signaling and transcriptional regulation, contrasting WT with IL-17A KO. **(G)** Scatter plot showing SCENIC regulon rankings in WT and IL-17A KO. **(H)** Heatmap showing differential transcription factor expression (SCENIC) between WT and IL-17A KO across neutrophil subclusters.

We further examined ligand-receptor interactions originating from endothelial cells, fibroblasts, osteoblasts, and neutrophils to neutrophils themselves. In WT mice, neutrophil communication was largely governed by two autocrine positive feedback loops. These loops involved Ccl3/Ccl6 signaling through Ccr1, and Cxcl2/Cxcl3 signaling through Cxcr2. Other enriched interactions in WT neutrophils involved Tnf–Tnfrsf1a/b, Spp1–Cd44, and Selplg–Sell. Cd47 signaling was also markedly increased in the absence of IL-17A, primarily through binding to Thbs1/2/3/4 (**Figure 5D**). NicheNet analysis was applied on same source cell types to evaluate which differential extracellular cues between WT and IL-17A KO groups regulate neutrophil transcriptional programs. Among all ligands, Tnf and Il1b were identified as the most influential differential ligands driving downstream key transcription factors and chemokine expression in neutrophils. Tgfb1 emerged as a ligand capable of specifically regulating Col1a1 and Thbs1 expression. Other influential ligands included Csf1, Il6, and Hgf (**Figures 5E, F**). SCENIC analysis indicated that transcription-factor activity in neutrophils was globally higher in WT than in IL-17A KO mice, including key regulators such as Atf3 and Fos (**Figure 5G**). The trends in transcription factor activity were broadly consistent across the different neutrophil clusters, with the most significant differences observed specifically in clusters 4 and 3 (**Figure 5H**).

## 4 Discussion

Although IL-17A is typically regarded as proinflammatory and proresorptive, its actions on bone are context dependent, with reports describing both osteoclast promoting and osteoblast supportive effects. In our *P. gingivalis* induced periodontitis model, IL-17A deficiency coincided with exacerbated bone loss. Together with micro-CT and TRAP evidence of active resorption in knockout mice, these observations indicate that osteoclastogenesis can proceed in the absence of IL-17A, likely via compensatory inflammatory pathways, and that loss of IL-17A may diminish osteoblast coupling signals. Mechanistically, IL-17A can influence osteoclasts both directly, by enhancing osteoclastogenic signaling and cooperating with inflammatory mediators, and indirectly, by increasing the RANKL/OPG ratio and shaping the inflammatory milieu, yet it may support osteoblast coupling in certain contexts. In our dataset, single cell ligand receptor inference did not reveal strong direct neutrophil to osteoclast interactions, noting the limited capture of mature multinucleated osteoclasts in droplet based assays. Building on these findings, we next considered how the loss of IL-17A interfaces with host–microbiota dynamics to influence tissue outcomes *in vivo*.

IL-17A deficiency is likely to perturb oral microbial homeostasis in addition to exacerbating tissue destruction. In this model, loss of IL-17A by impairing neutrophil recruitment and reshaping neutrophil transcriptional states would be expected to weaken host containment of dysbiosis, thereby linking immune tone to community structure (6–10). Consistent with this framework, 16S rRNA sequencing revealed IL-17A-dependent shifts in overall community composition, with enrichment of *Porphyromonas* and *Bacteroides* and reduced *Staphylococcus*, *Lactobacillus*, and *Olsenella* in IL-17A-deficient mice. While *P. gingivalis* is closely linked to periodontitis and oral immune regulation, periodontitis is polymicrobial rather than *P. gingivalis*-dependent alone, and community-level changes can arise from altered host immunity (30, 31). Accordingly, we considered both *P. gingivalis* and broader genus-level trends to capture ecosystem-scale effects of IL-17A deficiency. Given these observations, we next examined how IL-17A loss reshapes neutrophil abundance and transcriptional states *in vivo* under a standardized microbial challenge.

In this context, neutrophils are the most abundant immune cell group in the periodontal pocket and the first line of defense against microbial infection (32–36). Consistent with previous reports, the scRNA-seq analysis showed that IL-17A deficiency markedly impaired neutrophil accumulation in periodontal tissues following live *P. gingivalis* challenge (14, 37, 38). However, the impact of IL-17A loss was not limited to a quantitative reduction in neutrophil recruitment. Rather, IL-17A deficiency was accompanied by extensive remodeling of neutrophil heterogeneity and functional state (39–44). In the present study, five transcriptionally distinct neutrophil subclusters were identified, each characterized by specific marker gene profiles. WGCNA further resolved four co-expression modules associated with core neutrophil programs, including granule exocytosis and microbicidal activity, pro-inflammatory cytokine and chemokine signaling, oxidative metabolism and tissue surveillance, as well as ribosomal activity and *de novo* protein synthesis. These transcriptional programs displayed genotype-dependent distribution patterns. Neutrophils from WT mice preferentially exhibited inflammatory amplification and biosynthetic programs, whereas neutrophils from IL-17A-deficient mice were relatively skewed toward antimicrobial granule-associated and metabolic states (20, 29). Notably, although antimicrobial module genes were transcriptionally enriched in the IL-17A-deficient condition, the absolute neutrophil abundance was markedly reduced, suggesting that this transcriptional shift may not be sufficient to compensate for impaired neutrophil recruitment and the resultant defect in periodontal host defense. However, functional consequences require direct testing.

Pseudotime and RNA velocity analyses further indicated that neutrophil heterogeneity in this model reflected dynamic functional state transitions rather than a simple linear developmental hierarchy. Inflammatory stimulation is known to induce emergency granulopoiesis, leading to the release of neutrophils with diverse maturation and activation states into the circulation (45–47). In line with this concept, our trajectory analysis revealed interconversion among functionally distinct neutrophil states in inflamed periodontal tissues (48). Ccl3^+^ neutrophils represented a highly activated inflammatory subset enriched for chemotaxis, neutrophil activation, oxidative stress, and reactive oxygen species–related programs, whereas Ltf^+^ and Retnlg^+^ neutrophils were positioned toward a granule-rich and innate immune response–associated state. The increase in Stat3 expression and STAT3 regulon activity along the trajectory further suggested that transcriptional regulation may contribute to neutrophil state remodeling under IL-17A-deficient conditions, whether this translates into altered effector function remains to be determined. Importantly, IL-17A deficiency reduced the abundance of several neutrophil subclusters, particularly Ccl3^+^ inflammatory and Tgfbi^+^ repair-associated neutrophils, while relatively enriching Ltf^+^ and Retnlg^+^ populations. These data indicate that loss of IL-17A not only diminishes neutrophil influx but also reshapes the balance among inflammatory, repair-associated, antimicrobial, and infiltrating neutrophil states.

In addition to regulating cell-intrinsic transcriptional programs, IL-17A deficiency substantially remodeled neutrophil-centered communication networks within the periodontal microenvironment. In WT mice, neutrophil interactions were dominated by CCL, CXCL, TNF, SELPG, and ICAM signaling, forming autocrine and paracrine circuits that may sustain neutrophil recruitment and inflammatory amplification. Specifically, Ccl3/Ccl6–Ccr1 and Cxcl2/Cxcl3–Cxcr2 interactions constituted prominent neutrophil autocrine feedback loops (49–51). In contrast, IL-17A deficiency shifted the communication landscape toward extracellular matrix-associated pathways, including COLLAGEN, THBS, LAMININ, and FN1 signaling. Increased Cd47 interactions with Thbs family ligands, together with enhanced ECM-related communication from fibroblasts, endothelial cells, and osteoblasts, suggested that the IL-17A-deficient periodontal microenvironment is characterized by augmented adhesion- and matrix-associated signaling (52–56). NicheNet analysis further supported this shift, showing that neutrophil transcriptional programs in WT mice were mainly linked to TNF and IL-1β, whereas IL-17A-deficient neutrophils were more closely associated with TGF-β/THBS-related regulatory circuits. These findings suggest that IL-17A deficiency attenuates inflammatory chemokine/cytokine communication while promoting an ECM-responsive neutrophil transcriptional state.

The deterioration of periodontal tissue destruction observed in IL-17A-deficient mice may also be related to disruption of oral microbial homeostasis. In this study, IL-17A deficiency altered the oral microbiota structure under sustained *P. gingivalis* challenge, with enrichment of taxa such as *Porphyromonas* and Bacteroides, accompanied by changes in community diversity and phylum-level composition. Given the reduced neutrophil accumulation and altered neutrophil functional programs in IL-17A-deficient mice, these microbial changes may reflect a weakened capacity to constrain dysbiotic expansion during periodontal infection. *P. gingivalis* is known to interact with host antimicrobial mechanisms, including neutrophil ROS generation, phagocytosis, and neutrophil extracellular trap formation (57–66). However, whether IL-17A deficiency directly impairs these neutrophil effector functions during chronic live *P. gingivalis* challenge remains to be determined. Future studies integrating functional assays of bacterial killing, ROS production, phagocytic capacity, and NET formation will be necessary to clarify how altered neutrophil states contribute to microbial dysbiosis and periodontal bone loss in the absence of IL-17A. However, whether IL-17A deficiency directly impairs these neutrophil effector functions during chronic live *P. gingivalis* challenge remains to be determined. While a direct effect of absent IL-17A signaling on neutrophils is plausible, indirect contributions from altered microbial antigen load cannot be excluded. Besides, the interpretations are transcriptional and will require functional follow-up. In future studies, we will pair scRNA-seq–defined neutrophil states with matched functional readouts (bacterial killing, ROS, phagocytosis, NETs) to directly test the predicted deficits under IL-17A deficiency.

Overall, this study demonstrates that IL-17A deficiency aggravates *P. gingivalis*–induced periodontitis and is associated with increased alveolar bone resorption, enhanced osteoclast accumulation, oral microbial dysbiosis, impaired neutrophil recruitment, and remodeling of neutrophil-centered transcriptional and communication networks. At single-cell resolution, our findings reveal that loss of IL-17A disrupts the balance of neutrophil functional states and shifts periodontal immune regulation from chemokine/cytokine-driven inflammatory circuits toward extracellular matrix- and TGF-β/THBS-associated signaling. These results provide new insight into the pathological consequences of IL-17A deficiency in dysbiotic periodontal inflammation.

## Data Availability

The datasets presented in this study can be found in online repositories. The names of the repository/repositories and accession number(s) can be found in the article/**Supplementary Material**.
