# Supplementary material for: Neutrophil heterogeneity under IL-17A deficiency in experimental periodontitis
Source: Front Immunol. 2026 Oct 13;17:1963694. doi: 10.3389/fimmu.2026.1963694 (PMC13710030; doi:10.3389/fimmu.2026.1963694)
Supplement: Supplementary file 1 [file DataSheet1.docx]

# Supplementary information

Neutrophil Heterogeneity under IL-17A Deficiency in Experimental Periodontitis

Zhongyuan Tang^1^, Renjie Hu^1^, Luying Zhu^1^, Jing Hu^1^, Fan Xiao^3^, Lijian Jin^2^, Liwei Lu^3^, Yanqi Yang^1*^

^1^Division of Paediatric Dentistry and Orthodontics, Faculty of Dentistry, The University of Hong Kong, Hong Kong SAR, China

^2^Division of Periodontology and Implant Dentistry, Faculty of Dentistry, The University of Hong Kong, Hong Kong SAR, China

^3^Department of Pathology and Shenzhen Institute of Research and Innovation, The University of Hong Kong, Hong Kong SAR, China

*** Correspondence:**
Yanqi Yang
yangyanq@hku.hk

**Supplementary Figure 1 Analyses of oral microbiota in mice with *P. gingivalis*-induced periodontitis.**

**(A)** Intersection diagram. **(B)** Comparison of the WT and IL 17 KO mice assessed through PCoA based on beta diversity analysis. **(C)** Observed species index, Chao index, ACE index, Shannon index, Simpson index, and good-coverage index values from alpha diversity analysis. **(D)** Heatmap showing differences in the relative abundance of the bacterial genera with the most notable discrepancies. **(E)** LEfSe analysis conducted to determine distinctive features that differentiate the two groups.

**Supplementary Figure 2 Neutrophil DEGs and intercellular signaling alterations in WT vs IL‑17A KO mice**

**(A)** Waterfall plot of average log2 fold‑changes for differentially expressed genes (DEGs) in neutrophils in healthy mice (WT vs IL‑17 KO). **(B)** Specific signaling changes by interaction strength in the cell populations between WT and IL‑17 KO mice with periodontitis.

**Supplementary Table 1** Top 10 marker genes of neutrophil subclusters.

| Cluster | Adjusted P | Gene | Cluster | Adjusted P | Gene |
| --- | --- | --- | --- | --- | --- |
| 0 | 0 | Il1a | **3** | 0 | Ltf |
|  | 0 | Ccl3 |  | 0 | Itgb2l |
|  | 0 | Tnf |  | 0 | Chil3 |
|  | 0 | Il1rn |  | 0 | Camp |
|  | 1.30E-259 | Csf1 |  | 0 | Serpinb1a |
|  | 4.39E-219 | Ccl4 |  | 0 | Ngp |
|  | 1.81E-157 | Clec4n |  | 0 | Abca13 |
|  | 5.26E-148 | Isg15 |  | 7.67E-306 | Lbp |
|  | 3.85E-137 | Cxcl3 |  | 1.79E-294 | Zmpste24 |
|  | 8.06E-55 | Rsad2 |  | 1.50E-288 | Ldhc |
| 1 | 5.97E-143 | mt-Nd4l | **4** | 0 | Ly6g |
|  | 1.95E-137 | mt-Nd2 |  | 0 | Ly6c2 |
|  | 3.44E-134 | mt-Nd3 |  | 0 | Mmp8 |
|  | 1.51E-122 | mt-Nd1 |  | 0 | Retnlg |
|  | 2.75E-122 | mt-Nd5 |  | 3.18E-120 | Ceacam10 |
|  | 3.90E-116 | mt-Cytb |  | 4.20E-120 | Atrn |
|  | 1.86E-110 | Gcnt2 |  | 2.26E-116 | Prok2 |
|  | 1.49E-75 | Impact |  | 3.73E-92 | Zdhhc3 |
|  | 4.25E-68 | Fosb |  | 1.40E-59 | Acvrl1 |
|  | 2.47E-60 | Gm2a |  | 3.30E-52 | Rab3d |
| 2 | 5.82E-97 | Ccl6 | **5** | 8.08E-40 | Dmkn |
|  | 3.30E-46 | Tgfbi |  | 2.39E-34 | Krt14 |
|  | 4.76E-32 | Jaml |  | 3.90E-31 | BC037156 |
|  | 8.05E-31 | Wfdc17 |  | 3.24E-14 | Fabp5 |
|  | 1.18E-29 | Cpd |  | 8.35E-11 | mt-Nd5 |
|  | 7.76E-23 | Timp2 |  | 1.36E-06 | Rps18 |
|  | 3.21E-21 | Rdh12 |  | 5.96E-05 | Rpl10a |
|  | 2.35E-17 | Cers6 |  | 0.322435 | Il1a |
|  | 3.62E-13 | Hist1h1c |  | 1 | Ccnl2 |
|  | 9.43E-13 | Gng2 |  | 1 | Zfp263 |

**Supplementary Table 2** Top 20 downregulated DEGs in neutrophils from healthy IL‑17 KO mice versus healthy wild‑type controls.

| Symbol | log2 Fold Change | P value |
| --- | --- | --- |
| AY036118 | -0.96068 | 4.22E-106 |
| Tnfrsf26 | -0.98763 | 1.78E-05 |
| Chst11 | -0.99009 | 7.43E-17 |
| Ccdc186 | -1.00185 | 9.01E-11 |
| mt-Cytb | -1.00995 | 9.34E-91 |
| Ralgds | -1.08965 | 1.93E-05 |
| Tmx4 | -1.09868 | 9.59E-18 |
| Hmox1 | -1.10099 | 5.42E-28 |
| Upp1 | -1.10244 | 3.19E-17 |
| mt-Nd4l | -1.10766 | 3.53E-18 |
| mt-Nd4 | -1.16454 | 2.68E-115 |
| Batf | -1.22405 | 6.22E-08 |
| B4galt5 | -1.23607 | 2.90E-21 |
| Prok2 | -1.28571 | 1.73E-15 |
| mt-Nd1 | -1.31131 | 1.26E-91 |
| mt-Atp8 | -1.31546 | 0.003861 |
| mt-Nd5 | -1.3256 | 5.18E-48 |
| Snhg5 | -1.3299 | 1.51E-06 |
| mt-Nd2 | -1.7272 | 7.45E-132 |
| Mt1 | -1.75401 | 2.31E-24 |

**Supplementary Table 3** Top 20 upregulated DEGs in neutrophils from healthy IL‑17 KO mice versus healthy wild‑type controls.

| Symbol | log2 Fold Change | P value |
| --- | --- | --- |
| Dcpp1 | 3.104016 | 1.06E-27 |
| Dcpp3 | 2.777259 | 1.18E-18 |
| Mpp7 | 2.46635 | 1.65E-123 |
| Col3a1 | 1.704142 | 5.61E-15 |
| Igkc | 1.435299 | 8.01E-69 |
| Sbpl | 1.431423 | 1.25E-31 |
| Mpo | 1.342857 | 1.05E-19 |
| Dcn | 1.31484 | 3.41E-08 |
| Elane | 1.291957 | 3.63E-42 |
| Slc15a2 | 1.28621 | 2.63E-11 |
| Prtn3 | 1.271829 | 2.15E-20 |
| Nupr1 | 1.247771 | 1.66E-43 |
| Ptgs2 | 1.225982 | 1.35E-15 |
| Mpzl3 | 1.183166 | 1.17E-08 |
| Taf1 | 1.119243 | 4.32E-08 |
| Dot1l | 1.104914 | 6.72E-15 |
| Trf | 1.081695 | 8.73E-23 |
| Muc19 | 1.075753 | 9.97E-09 |
| Tff2 | 1.045971 | 8.24E-87 |
| Cd300lb | 1.03565 | 4.51E-20 |

**Supplementary Table 4** Top 20 downregulated DEGs in neutrophils from IL‑17 KO mice with periodontitis versus healthy wild‑type mice with periodontitis.

| Symbol | log2 Fold Change | P value |
| --- | --- | --- |
| AC149090.1 | -0.949 | 3.36E-17 |
| Isg20 | -0.99244 | 3.60E-52 |
| Rtp4 | -1.02393 | 3.09E-32 |
| Gm20536 | -1.03335 | 1.85E-25 |
| Eif4ebp1 | -1.04162 | 6.09E-50 |
| Lrg1 | -1.04828 | 2.34E-159 |
| Pycard | -1.0894 | 4.01E-19 |
| Parp14 | -1.09834 | 2.24E-20 |
| Gbp2 | -1.11058 | 1.99E-25 |
| Slfn5 | -1.17419 | 1.16E-30 |
| Tspo | -1.17844 | 1.77E-126 |
| Gm13822 | -1.2106 | 1.25E-22 |
| Irf7 | -1.22543 | 2.58E-28 |
| Igfbp6 | -1.26466 | 2.08E-46 |
| Ubb | -1.27164 | 0 |
| AY036118 | -1.41192 | 4.18E-119 |
| Ifitm1 | -1.43582 | 1.74E-267 |
| Wfdc17 | -1.54855 | 0 |
| Cxcl1 | -1.66727 | 3.53E-45 |
| Gm17056 | -2.83049 | 2.03E-85 |

**Supplementary Table 5** Top 20 upregulated DEGs in neutrophils from IL‑17 KO mice with periodontitis versus healthy wild‑type mice with periodontitis.

| Symbol | log2 Fold Change | | P value |
| --- | --- | --- | --- |
| Mpp7 | | 2.357704 | 2.50E-247 |
| Cdadc1 | | 1.870702 | 5.81E-33 |
| Igkc | | 1.827768 | 1.79E-86 |
| Adpgk | | 1.577353 | 5.23E-51 |
| Itgb2l | | 1.405093 | 1.41E-25 |
| 9830107B12Rik | | 1.379904 | 2.11E-58 |
| Hba-a2 | | 1.37199 | 7.07E-186 |
| Nt5e | | 1.354035 | 2.37E-17 |
| Hba-a1 | | 1.352763 | 2.76E-213 |
| Thbs1 | | 1.34332 | 2.39E-127 |
| 1700047M11Rik | | 1.315467 | 3.39E-11 |
| Cybb | | 1.274852 | 1.35E-34 |
| Man1b1 | | 1.202216 | 7.17E-26 |
| Krt83 | | 1.175851 | 5.64E-11 |
| Ankrd13c | | 1.096701 | 5.42E-10 |
| Cd93 | | 1.090387 | 5.58E-14 |
| Cd177 | | 1.08347 | 6.17E-38 |
| Mospd1 | | 1.064294 | 3.15E-06 |
| Ckap4 | | 1.063285 | 8.31E-39 |
| Pqlc3 | | 1.062838 | 6.12E-12 |

**Supplementary Table 6** Alpha diversity between WT and IL-17A KO group.

| **Alpha diversity** | **Mean (WT)** | **SD(WT)** | **Mean (KO)** | **SD (KO)** | **p-vaule** |
| --- | --- | --- | --- | --- | --- |
| Observed species | 199.3333 | 21.38535 | 219.6667 | 10.11599 | 0.2 |
| chao | 231.8068 | 9.96118 | 235.0439 | 6.03131 | 0.7 |
| ace | 234.7661 | 1.20929 | 233.8252 | 3.50957 | 0.7 |
| shannon | 2.39373 | 0.05229 | 2.51875 | 0.3254 | 0.7 |
| simpson | 0.18773 | 0.01429 | 0.21591 | 0.08067 | 0.7 |
| coverage | 0.99889 | 0.00047 | 0.99931 | 0.00053 | 0.4 |

**Supplementary Table 7** Relative abundance of genera between WT and IL-17A KO group.

| **Taxa** | **WT** | **KO** | **Taxa** | **WT** | **KO** |
| --- | --- | --- | --- | --- | --- |
| Pasteurella | 1.691011 | 1.653179 | Streptococcus | -0.17592 | -0.56402 |
| Escherichia | 0.348629 | 1.193918 | GpI | 0.047241 | -0.57394 |
| Lactobacillus | 1.125496 | 0.696824 | Clostridium_sensu_stricto | -0.83486 | -0.57826 |
| Salmonella | 0.815608 | 0.655838 | Blautia | -0.94636 | -0.62724 |
| Porphyromonas | 0.191625 | 0.598606 | Acetatifactor | -0.93589 | -0.72092 |
| Olsenella | 0.78236 | 0.550465 | Anaerostipes | -1.55914 | -0.72294 |
| Enterococcus | 0.327162 | 0.471607 | Flavonifractor | -0.81095 | -0.73523 |
| Bacteroides | -0.17116 | 0.216928 | Parasutterella | -1.07722 | -0.77662 |
| Corynebacterium | 0.209991 | 0.086385 | Roseburia | -1.04949 | -0.79056 |
| Allobaculum | 0.141852 | 0.046712 | Eubacterium | -1.23692 | -0.86225 |
| Clostridium_XlVa | -0.18074 | 0.036991 | Helicobacter | -1.19741 | -0.90309 |
| Chryseobacterium | -0.33903 | 0.016504 | Oscillibacter | -1.17893 | -0.92184 |
| Kosakonia | 0.140408 | -0.0128 | Lachnospiracea_incertae_sedis | -1.17893 | -0.92504 |
| Bifidobacterium | -1.31199 | -0.03709 | Alistipes | -1.21672 | -0.97259 |
| Staphylococcus | 0.744632 | -0.08041 | Actinobacillus | -1.14977 | -0.97983 |
| Parabacteroides | -0.89954 | -0.23452 | Lachnospira | -1.65605 | -1.01006 |
| Prevotella | -0.35412 | -0.26934 | Klebsiella | -1.4342 | -1.01399 |
| Barnesiella | -0.50351 | -0.28822 | Gemmiger | -1.07247 | -1.02599 |
| Faecalibacterium | -0.63314 | -0.30027 | Megamonas | -0.76675 | -1.25604 |
| Burkholderia | -0.49344 | -0.35467 | Enterorhabdus | -1.06778 | -1.43213 |
| Desulfovibrio | -0.77381 | -0.5239 | Other(<0.0005) | -1.39281 | -0.82238 |

Data are presented as the mean.
